# Near-field electron ptychography using full-field structured illumination

**DOI:** 10.1093/jmicro/dfae035

**Published:** 2024-07-25

**Authors:** Hirokazu Tamaki, Koh Saitoh

**Affiliations:** Graduate School of Engineering, Nagoya University, Furo-cho, Chikusa-ku, Nagoya, Aichi 464-8603, Japan; Research & Development Group, Hitachi Ltd., 1-280, Higashi-koigakubo, Kokubunji, Tokyo 185-8601, Japan; Institute of Materials and Systems for Sustainability, Nagoya University, Furo-cho, Chikusa-ku, Nagoya, Aichi 464-8603, Japan

**Keywords:** structured illumination, wavefield reconstruction, ptychography, in-line holography, phase measurement

## Abstract

A new configuration for near-field ptychography using a full-field illumination with a structured electron beam is proposed. A structured electron beam illuminating the entire field of view is scanned over the specimen, and a series of in-line holograms formed in the near-field region below the specimen are collected. The structured beam is generated by a conductive film with random openings, which ensures high stability and coherence of the beam. Observation in the near-field region reduces the beam concentration that occurs in the far-field region, which contributes to accurate recording of the beam intensity with a finite dynamic range of the detectors. The use of full-field illumination prevents the accumulation of errors caused by concatenating the local structures, which is the method used in conventional reconstruction. Since all holograms are obtained from the entire field of view, they have uniform multiplicity in terms of specimen information within the field of view. This contributes to robust and efficient reconstruction for a large field of view. The proposed method was tested using both simulated and experimental holograms. For the simulated holograms, the reconstruction of the specimen transmission function was achieved with an error less than 1/3485 of the wavelength. The method was further validated using experimental holograms obtained from MgO particles. The reconstructed phase transmission function of the specimen was consistent with the specimen structure and was equivalent to a mean inner potential of 13.53±0.16 V on the MgO particle, which is in close agreement with previously reported values.

## Teaser

A new configuration for near-field ptychography using full-field illumination with a structured electron beam is proposed. It enables robust reconstruction of large field of view. Simulation study showed phase reconstruction within an error of 1/3485 of the wavelength. Experimental study using MgO crystals also showed reasonable phase reconstruction.

## Introduction

The coherent diffractive imaging (CDI) is a technique to reconstruct the complex wavefield from far-field diffraction pattern. CDI has been rapidly advancing in recent years due to improvements in detector and computing performance and has been applied to X-ray and electron beam imaging [1–7]. One such method, called ptychography [8–10], has been attracting much interest in recent times.

Conventional ptychography involves collecting a series of diffraction patterns from local areas on the specimen, where the illumination areas overlap each other. The complex transmission function of the specimen is reconstructed by iterative calculation, which imposes constraints given by the illumination area overlaps. Overlap-based constraint is easy to introduce in comparison to focal series-based constraint, as it does not require any modifications to the lens systems, which have non-linearity due to magnetic hysteresis. Moreover, overlap-based constraint enables the observation area to be extended by simply increasing the number of diffraction patterns, and it also contributes to robust reconstruction against noise [11].

Conventional CDI is hampered by the wide intensity range of the far-field diffraction patterns. The far-field diffraction pattern usually includes strong signals such as Bragg peaks and weak signals such as Airy patterns and sample shape factors around the Bragg peaks. The intensity range of these features exceeds ${10^5}$, far beyond the dynamic range of conventional cameras. High dynamic range image synthesis from multiple images with different exposure times [3,12] or specially designed detectors [13] has been proposed for accurately recording far-field diffraction patterns.

Stockmar et al. [14] recently proposed a technique called near-field ptychography in the field of X-ray imaging. This technique uses Fresnel diffraction patterns observed in the near-field diffraction region or in-line holograms instead of far-field diffraction patterns. To increase the diversity of holograms, they introduce a diffuser that scrambles the incident beam. They reported that observation in the near-field region noticeably reduces the intensity range of the patterns, and the diffuser improves the signal-to-noise ratio of the reconstructed transmission function. Allars et al. [15] applied near-field ptychography to electron beams. They placed an ion-beam-fabricated silicon-nitride membrane on the selected-area aperture of transmission electron microscope (TEM), and acquired holograms with the field of view restricted by the selected-area aperture. Reconstruction is performed using multiple holograms collected by moving the specimen position relative to the beam.

In this study, we propose a new configuration for near-field ptychography for electron beams. It utilizes full-field and structured illumination generated by a conductive film with random openings. The proposed method is tested by using a series of simulated holograms and examined the accuracy and robustness of the reconstruction. The method was then applied to an experimental data set obtained by using a conventional TEM equipped with a 200 kV cold field emission gun.

## Methods

**Fig. 1.**
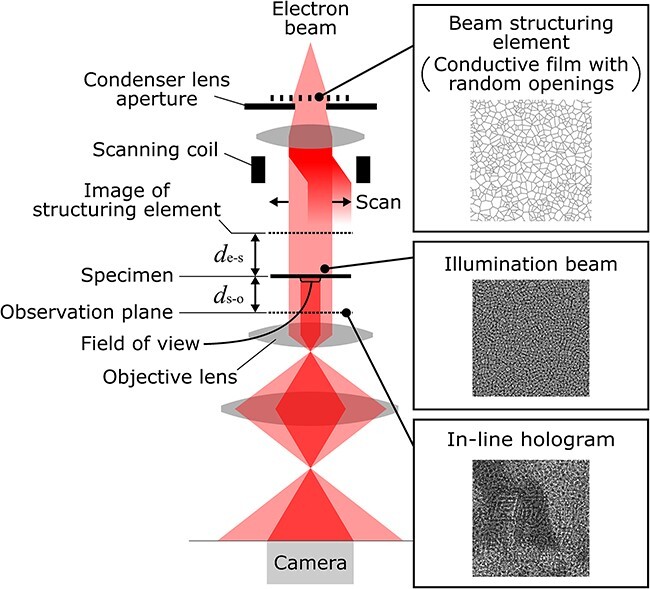
Schematic of electron optical system used for this study. Some of the condenser and imaging lenses are omitted for simplicity; ${d_{{\mathrm{e}} - {\mathrm{s}}}}$ and ${d_{{\mathrm{s}} - {\mathrm{o}}}}$ represent the optical propagation distance from the structuring element to the specimen and from the specimen to the observation plane, respectively.

Figure 1 shows the configuration of the electron optical system used in this study. A conductive film with random openings, which we call a ‘beam structuring element’ hereafter, is placed on the condenser lens aperture. The film’s random structure imparts amplitude and phase changes to the incident beam. The structure of the beam further alters as it propagates through the condenser lens system. The structured beam illuminates the specimen and undergoes amplitude and phase changes on the specimen transmission. Then, the transmitted beam further propagates and forms an interference pattern, an ‘in-line hologram’, in the near-field diffraction region below the specimen (shown as ‘Observation plane’ in the figure). The illumination area on the specimen is adjusted by the condenser lens system to cover the entire field of view which is recorded by the camera. The illumination beam is then scanned across the specimen by using the scanning coil above the specimen. A series of in-line holograms are collected from each respective illumination position. Then, the complex functions of both the specimen and illumination beam are reconstructed through a ptychographic reconstruction procedure.

The proposed method differs from the previous near-field electron ptychography in the use of full-field and structured illumination generated by a conductive film with random openings. Full-field illumination prevents error accumulation when reconstructing a large field of view compared with conventional ptychographic reconstruction, which ‘concatenates’ small patches of the local structures. In conventional ptychography, which overlaps the local illuminations, the multiplicity of specimen information among the holograms is not uniform within the region of interest. This non-uniformity is related to factors such as the degree of overlap of each local illumination, the arrangement of illumination positions, and the total number of holograms. In contrast, with full-field illumination, all the holograms are acquired from the whole region of interest, and thus the multiplicity of the specimen information is uniform for the whole region of interest. This suppresses excessive observation in certain areas on the specimen and improves the efficiency of data collection, also contributing to the reduction of electron dose to the specimen. Moreover, the size of the region of interest and the multiplicity of specimen information can be independently controlled by the magnification of the imaging system and the number of holograms, respectively. This enables efficient data collection and simple operation in the same manner as a conventional TEM.

The structured illumination provides a unique change in each hologram when the illumination beam is shifted. This provides an alternative constraint to the conventional constraint based on the overlap of local illuminations. Using a conductive film prevents the structuring element from charging during hologram acquisition. This suppresses fluctuations in the illumination beam structure, which is assumed to remain invariant in ptychographic reconstruction. Using the random openings for beam structuring is also beneficial as it minimizes beam coherence degradation caused by inelastic scattering from the structuring element. The structure of the illumination beam can be magnified or demagnified with the condenser lens system and can be further modulated by changing the effective propagation distance between the structuring element and the specimen, ${d_{{\mathrm{e}} - {\mathrm{s}}}}$.

The reconstruction was performed by coupling each plane by free space propagation using the angular spectrum method [16]. Ptychographic reconstruction was performed by iterative calculation using the ePIE algorithm [17]. The amplitude and phase for the iterative reconstruction were initially set to a random value for both the illumination beam and the specimen transmission functions.

Simulation studies were performed by using holograms calculated at an acceleration voltage of 200 kV. A Voronoi diagram was used to design irregular openings in the structuring element. Random seed points were prepared to correspond to the position of each opening, and bisector lines were drawn between each pair of neighboring points to generate the Voronoi diagram. Different numbers of seed points (275, 550, and 1100) were tested and each condition showed no observable differences in the reconstruction. We used 550 seed points for the present study.

The amplitude transmission rate and phase shift on the film were set to 99.9% and 2.0 rad, respectively, on the basis of the assumption of a 50-nm-thick carbon film. The structures of the amplitude and phase components of the specimen transmission function were set to be completely different. The distances from the specimen to the image of the structuring element above the specimen, ${d_{{\mathrm{e}} - {\mathrm{s}}}}$, and from the specimen to the observation plane below the specimen, ${d_{{\mathrm{s}} - {\mathrm{o}}}}$, were both set to 0.2 mm. The three planes (the structuring element plane, the specimen plane, and the observation plane) were each coupled at the same scale with a magnification of 1. The specimen structure was prepared for a 2.048 × 2.048 μm region and sampled with a pixel resolution of 1024 × 1024. To correctly incorporate the effects of diffracted waves from outside the field of view, in-line holograms were acquired from the central region of the specimen, which had a size of 1.024 × 1.024 μm. Holograms were calculated without taking into account the influence from the aberrations in the optical system and spatial coherence due to the finite electron source size. Two hundred different illumination positions were used to calculate a series of in-line holograms, which were randomly arranged within a horizontal and vertical range of ±80 nm. Iterative reconstruction was performed on each of the 200 holograms for one cycle, and repeated for 50 000 cycles. In the reconstruction process, the specimen and illumination beam structures were expanded by a factor of 1.25 to the acquired hologram in both width and height by padding with random-value regions. This ensured that the entire structure of the illumination beam involved in the generation of each hologram was correctly incorporated. Reconstruction accuracy was evaluated by measuring the error of the amplitude and phase component of the reconstructed specimen transmission function in the central and half-size area of the field of view.

Experimental examination was performed with a conventional 200 kV TEM equipped with a cold field emission electron source. A lacey carbon film with hole diameters of 1–4 μm mounted on the condenser lens aperture plate was used as a structuring element. The specimen used was composed of MgO particles dispersed on the carbon film. In the near-field region below the specimen, an in-line hologram was recorded without using an objective lens aperture. The field of view was set to 2.009 × 2.009 μm. Holograms were recorded using a 16-bit Gatan OneView CMOS camera with 4096 × 4096 pixels and an exposure time of 2.0 s, and then binned to 512 × 512 pixels. The electron dose rate on the specimen plane was 8.9 ${{\mathrm{e}}^ - }/{{\unicode{x00C5}}^2}$, resulting in a total electron count of $3.6 \times {10^9}$ for a single hologram. A total of 81 in-line holograms were obtained by shifting the illumination beam position by nine steps with an interval of 67 nm in two directions almost orthogonal to each other. The deflection ratio of the upper and lower scanning coils was adjusted so that no tilt change occurs during the illumination beam shift. This was carried out by minimizing the displacement of the in-line hologram induced by the beam shift of the plane wave illumination. In this study, the deflection ratio was adjusted so that the displacement of the in-line hologram is less than 2 pixel during the illumination beam shift. As a result, beam tilt due to the illumination beam shift was suppressed within ±$2.4\times10^{-7}$ rad, which corresponds to the phase change of the illumination beam less than ±$0.010$ rad in the region of single MgO particle with 100-nm diameter. This effect can be considered to have an insignificant effect for the specimen scale of this study. The condenser lens system was adjusted so that the illumination beam covered the entire field of view. The propagation distance from the structuring element to the specimen (${d_{{\mathrm{e}} - {\mathrm{s}}}}$ in Fig. 1) was adjusted so that the Fresnel fringes covered the holes of the structuring element and no flat intensity regions were present in the illumination beam on the specimen plane. The two-fold astigmatism and current-centering axis for the objective lens were initially adjusted in the in-focus condition to the specimen using the conventional adjustment procedure of TEM. Then, the propagation distance from the specimen to the observation plane (${d_{{\mathrm{s}} - {\mathrm{o}}}}$ in Fig. 1) was controlled mainly by moving the specimen position along the optical axis, and adjusted supplementally by the objective lens focus. To ensure the recording of the wave propagation from the specimen plane, the propagation distance ${d_{{\mathrm{s}} - {\mathrm{o}}}}$ was adjusted based on the size of the first Fresnel zone ${r_{{\mathrm{FZ}}1}}$, which is given by Eq. (1):


(1) $${r_{{\mathrm{FZ}}1}} = \sqrt {\lambda {d_{{\mathrm{s}} - {\mathrm{o}}}}} \\[1.3pt]$$

where λ represents the wavelength. The propagation distance ${d_{{\mathrm{s}} - {\mathrm{o}}}}$ was chosen such that the ${r_{{\mathrm{FZ}}1}}$ exceeds several times the pixel size on the observation plane.

The propagation distance ${d_{{\mathrm{s}} - {\mathrm{o}}}}$ was calibrated in the reconstruction process to a value of 1.54 mm, at which the reconstructed specimen amplitude showed no Fresnel fringes at the edge of the largest particle. Iterative reconstruction was performed on each of the 81 holograms for one cycle, and repeated for 5000 cycles. The holograms were acquired with an angular spread of the electron beam $\beta $ of $2.1 \times {10^{ - 6}}$ rad, which represents the degree of spatial coherence originating from the finite size of the electron source [18]. This angular spread leads to disappearance of fine fringes in the interference pattern, affecting both the illumination beam pattern and the in-line holograms. For the illumination beam, the influence can be considered as a change in the wavefield and can be separated from the specimen information through ptychographic reconstruction. However, the disappearance of the fringes in the in-line hologram could reduce the spatial resolution in the reconstruction of specimen information. The spatial resolution in the reconstruction can be estimated as $d$, which is defined by Eq. (2) [18], where $L$ represents the propagation distance from the specimen to the observation plane.


(2) $$d = 3.2\beta L$$

The estimated value of $d$ is approximately 10.3 nm. This value is close to the resolution limit of 7.8 nm, which was calculated based on the Nyquist frequency of the hologram. This indicates that the spatial coherence has a limited influence under the conditions of this experiment and specimen scale.

## Results and discussion

**Fig. 2.**
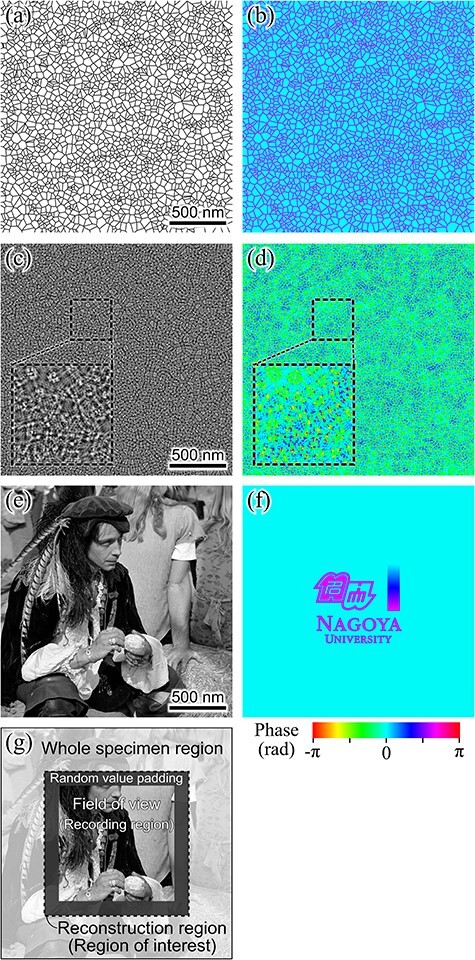
Given structures for simulation study. (a), (b) Amplitude and phase components of transmission function of structuring element. Amplitude transmission rate ranged from 99.9% to 100%. (c), (d) Amplitude and phase of illumination beam at specimen plane. (e), (f) Amplitude and phase components of transmission function of specimen. Amplitude transmission rate ranged from 50% to 100%. (g) Relationship among the whole specimen region, field of view, padding region, and reconstruction region.

Figure 2a and 2b, respectively, show the amplitude and phase of the transmission function of the structuring element used in the simulation study. The white and black regions in the amplitude image represent the openings and the film, respectively. Changes in the amplitude and phase of the illumination beam occurred in the film region. Figure 2c and 2d, respectively, show the amplitude and phase of the illumination beam after passing through the film and propagating a distance of 0.2 mm. Fresnel fringes filled the openings of the film, forming an entirely random structure with no flat regions. Figure 2e and 2f, respectively, show the amplitude and phase components of the specimen transmission function used in the simulation study.

**Fig. 3.**
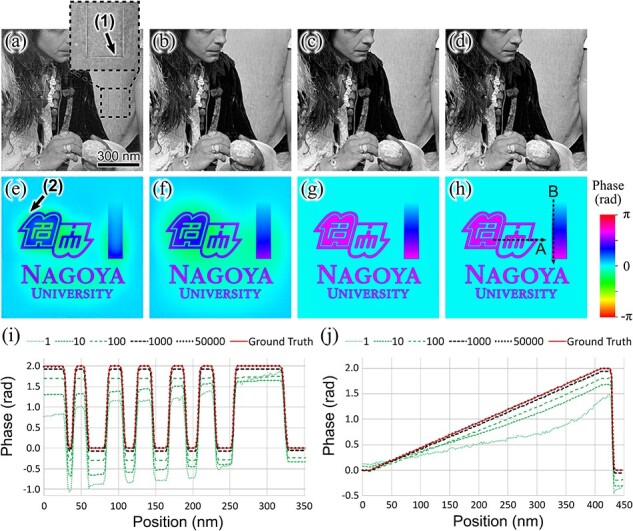
Reconstructed transmission function and given structure of specimen. (a), (e) Reconstructed amplitude and phase components at first cycle. (b), (f) Reconstructed amplitude and phase components at fifth cycle. (c), (g) Reconstructed amplitude and phase components at 50 000th cycle. (d), (h) Amplitude and phase components of given structure (ground truth). (i) Phase profile obtained from reconstructed phase component at each iteration cycle and ground truth along line A in (h). (j) Phase profile obtained from reconstructed phase component at each iteration cycle and ground truth along line B in (h).

Figure 3a–c and 3e–g, respectively, show amplitude and phase of the specimen transmission functions reconstructed from the 200 simulated holograms with the 1, 5, and 50 000 reconstruction cycles. Figure 3d and 3h, respectively, show the amplitude and phase components of the given specimen structure (ground truth) obtained from the corresponding region.

The amplitude component at the first cycle (Fig. 3a) shows a rectangular artifact, as indicated by arrow (1). It apparently originated from the phase component. This artifact gradually faded away as the iteration cycle progressed and disappeared by the 50 000th cycle. The phase component at the first cycle (Fig. 3e) had a spreading halo around the original structures, as indicated by arrow (2). This artifact gradually spread out and converged to a flat phase as the iteration cycle progressed. The transmission function reconstructed at the 50 000th cycle shows good agreement with the ground truth, with an average error of 0.051% in amplitude and 0.0036 rad in phase.

Figure 3i and 3j show line profiles of the phase reconstructed at the first, 10th, 100th, 1000th, and 50 000th reconstruction cycle and the ground truth taken along black dotted lines A and B in Fig. 3h, respectively. The rectangular structures of the phase along line A were approximately reconstructed from the first iteration cycle. As the iteration cycle progressed, the phase between the sharp edges approached the flat ground truth. Note that the positions of the sharp edges were accurately reconstructed from the first iteration cycle. Similarly, the sharp edge of the phase profile along line B was reconstructed from the first cycle. The slope of the phase profile gradually approached the ground truth as the iteration cycle progressed. The maximum phase errors of the reconstructed profiles were 0.00052 rad for the single rectangular structure in line A from 160 to 200 nm and 0.0018 rad for the entire slope in line B. The errors were approximately 1/12 097 and 1/3485 of the wavelength, respectively, which are comparable to those of other methods used for accurate phase measurement [19].

The proposed method reconstructed the phase more accurately in specimen regions containing fine structures, such as in line A, than in regions containing coarse structures, such as in line B. This is thought to be related to the amount of illumination beam shift with respect to the size of the structure of interest on the specimen. Solving the inverse problem for reconstruction requires holograms with different illumination conditions. Therefore, the shift amount of the illumination beam should be larger than the size of the structure of interest on the specimen. In the present simulation study, the amount of illumination beam shift is larger for the structure in line A and smaller for the structure in line B. Therefore, it is assumed that this has resulted in lower reconstruction accuracy for line B. The suitable amount of the beam shift should be determined on the basis of the specimen structure, as was noted in a previous study [20].

The Fresnel number $F$ [21], an index corresponding to the number of Fresnel zones for a wave passing through a certain area and propagating from the specimen plane to the destination plane, is expressed by the following Eq. (3).


(3) $$F = \frac{{{a^2}}}{{L\lambda }}$$

where $a$, $L$, and $\lambda $ represent the size of the area on the specimen, the propagation distance, and the wavelength, respectively. Fresnel number $F$ also corresponds to the fineness of an interference pattern formed by a wave passing through an area with a certain size. $F$ is calculated to be 319 for the large slope structure in the specimen. This large Fresnel number indicates that the interference pattern from the large area of the slope structure forms fine fringes. These fringes are relatively difficult to resolve due to the limited pixel resolution of the hologram. The fineness of the interference pattern, which depends on the size of the specimen structure, is considered to be one of the reasons for the lower phase reconstruction accuracy in the large slope structure.

**Fig. 4.**
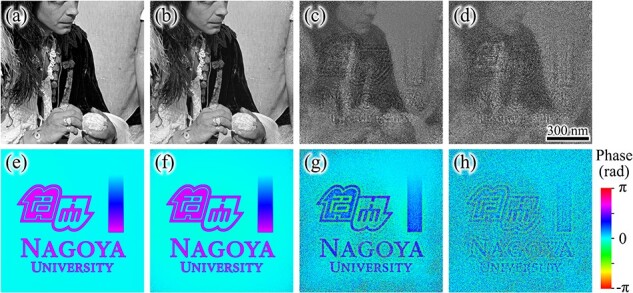
Specimen transmission functions in field of view reconstructed from different numbers of in-line holograms. (a)–(d) Amplitude component of reconstructed transmission function using 16, 9, 8, and 4 holograms, respectively. (e)–(h) Phase component of reconstructed transmission function using 16, 9, 8, and 4 holograms, respectively.

Reconstruction was also tested using a structuring element that completely blocks the beam. The reconstructed specimen transmission function showed almost the same value as that reconstructed using the transparent element in the previous test, with an average error of 0.062% in amplitude and 0.0065 rad in phase to the ground truth. This indicates that reconstruction can be performed regardless of whether the structuring element is based on the phase change or the amplitude change. Figure 4a–d and 4e–h show amplitude and phase of the specimen transmission function reconstructed from a total of 16, 9, 8, and 4 holograms. Each reconstruction is performed using the transparent structuring element mentioned above, with a total of 50 000 iteration cycles. The average errors of the reconstructed phase were 0.0040, 0.010, 0.56, and 0.83 rad when the total number of holograms was 16, 9, 8, and 4, respectively. The average phase error increased substantially when the number of holograms was reduced from nine to eight. When the total number of holograms was eight or less, the reconstructed phase showed an increase in noise and caused an artifact due to the crosstalk from the amplitude structure. This indicates that nine or more holograms were needed for proper reconstruction, in the case studied here. The minimum number of holograms required for near-field ptychography is considered to be four to satisfy the oversampling condition [20]. This is because two complex functions (those for the illumination beam and specimen, which each have 2*N* unknown numbers) must be reconstructed from the intensities of the holograms. Each hologram has *N* known numbers, where *N* is the total number of pixels in the image. However, the required number is more than four in actuality. This is apparently because each hologram is formed using a different area of the illumination beam. In the present case, the reconstruction is performed in an area 1.56 ($ = {1.25^2}$) times larger than the field of view to include all of the structure of the illumination beam used in the generation of each hologram. This indicates that more holograms are required to satisfy the oversampling condition. It should be noted that the requirement of nine holograms is small enough compared to the conventional ptychographic reconstruction, and is effective to reduce the total time of data collection.

**Fig. 5.**
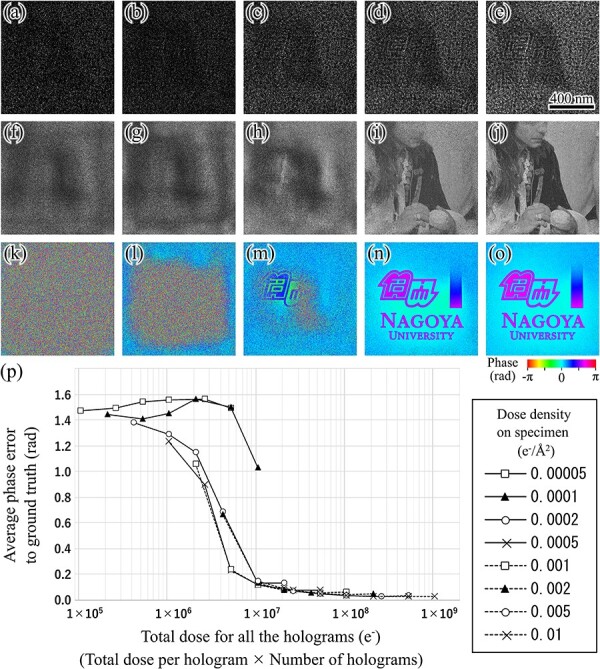
Reconstruction from in-line holograms with different amounts of noise. (a)–(e) Example holograms with different amounts of noise corresponding to dose densities of 0.00005, 0.0001, 0.0002, 0.0005, and 0.001 ${{\mathrm{e}}^ - }/{{\unicode{x00C5}}^2}$, respectively. (f)–(j) Amplitude components of specimen transmission function reconstructed under noise condition corresponding to (a)–(e), respectively. (k)–(o) Phase components of specimen transmission function reconstructed under noise condition corresponding to (a)–(e), respectively. (p) Variation in average error in reconstructed phase of specimen function with respect to total dose for all the holograms (total dose per hologram × number of holograms used for reconstruction).

Figure 5a–e show holograms with Poisson noise corresponding to electron dose densities of 0.00005, 0.0001, 0.0002, 0.0005, and 0.001 ${{\mathrm{e}}^ - }/{{\unicode{x00C5}}^2}$, respectively. Figure 5f–j and 5k–o show the amplitude and phase components of the specimen transmission functions reconstructed from 200 holograms simulated under dose densities of 0.00005, 0.0001, 0.0002, 0.0005, and 0.001 ${{\mathrm{e}}^ - }/{{\unicode{x00C5}}^2}$, respectively. The reconstruction was performed with 5000 iteration cycles. The specimen structure was properly reconstructed from the holograms with a small amount of noise, as shown in Fig. 5d and e. In contrast, the reconstructed specimen transmission function loses the fine structure in the higher noise condition, as shown in Fig. 5c, and no longer exhibits a visible structure in the highest noise condition, as shown in Fig. 5a and b, which corresponds to a dose amount below 0.0001 ${{\mathrm{e}}^ - }/{{\unicode{x00C5}}^2}$. Figure 5p shows the average error in the phase of the reconstructed specimen transmission function as a function of the total dose amount for all the holograms used for the reconstruction. The total dose amount is given by the product of the total electron count for single hologram and the number of holograms used for reconstruction. Poisson noise is introduced into the holograms in accordance with the dose intensity. Iterative reconstruction was performed until the product of the number of holograms and the iteration cycle reached 1 000 000. At dose levels below 0.0001 ${{\mathrm{e}}^ - }/{{\unicode{x00C5}}^2}$, average phase error does not show a substantial change to the increase of the total number of holograms. This indicates that a certain amount of signal is required for a single hologram to perform the reconstruction. At dose levels above 0.0002 ${{\mathrm{e}}^ - }/{{\unicode{x00C5}}^2}$, the error decreases with the total number of holograms increases, and are distributed along a common curve as a function of the total dose for all the holograms. This indicates that the phase accuracy is related to the total number of electrons all the holograms used for the reconstruction. The total number of holograms should thus be adjusted on the basis of the current density of the illumination beam. In this case, reconstruction is performed with an average phase error below 0.1 rad with the total dose for all the holograms over $1 \times {10^8}$.

**Fig. 6.**
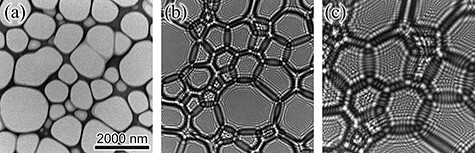
Images of the structured beam with different amounts of defocus (propagation distance) from the structuring element. The scale bar corresponds to the scale on the specimen plane. (a) Focused to the element; (b) moderately defocused; (c) highly defocused.

The proposed method was applied to experimental holograms obtained from MgO particles. Figure 6a–c show images of the illumination beam on the specimen plane, observed under in-focus, moderately defocused, and highly defocused conditions, respectively, with respect to the structuring element. Fresnel fringes appear from the edge of the lacey film with defocusing and fill each opening of the film entirely in the highly defocused condition, as shown in Fig. 6c. The illumination beam pattern, as shown in Fig. 6c, is employed in the experimental study. This pattern is subsequently scanned over the MgO particle for the collection of in-line holograms. The electron dose for a single hologram is $3.6 \times {10^9}$ electrons, and $2.9 \times {10^{11}}$ electrons in total for the 81 holograms, which is much higher than the dose rate required for achieving the phase reconstruction accuracy of less than 0.1 rad for 512 × 512 pixel region, as shown in Fig. 5.

**Fig. 7.**
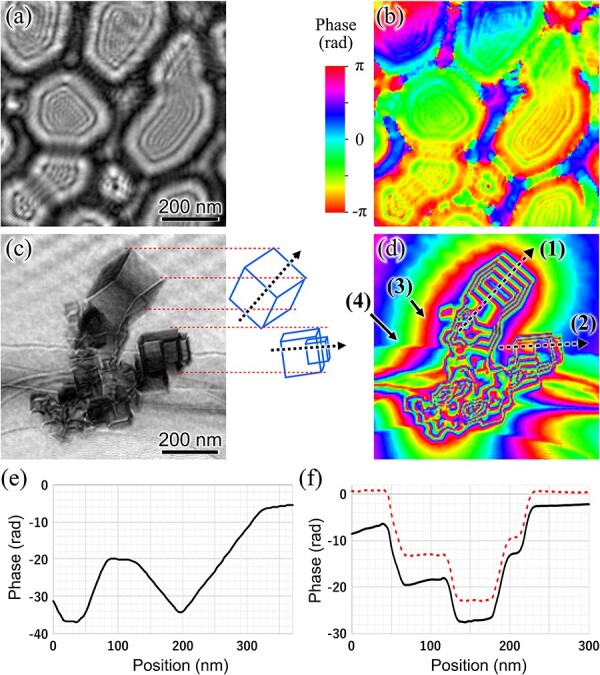
Reconstructed illumination beam wavefield and specimen transmission function. (a), (b) Amplitude and phase of illumination beam wavefield, respectively. (c), (d) Amplitude and phase of specimen transmission function, respectively. (e), (f) Phase profile of unwrapped phase taken from reconstructed specimen transmission function along lines (1) and (2), respectively, in (d). Red dashed line in (f) shows a phase profile along line (2) after subtracting a background phase gradient obtained from the adjacent vacuum region.

Figure 7a and b show the amplitude and phase of the reconstructed illumination beam, respectively; 808 × 808 nm region around the MgO particle is shown in this figure. The amplitude of the illumination beam shows a structure composed of lacey film and Fresnel fringes, which is similar to that observed in Fig. 6c. This indicates that this method reasonably reconstructed the illumination beam wavefield. Figure 7c shows an amplitude component of the reconstructed specimen transmission function. The amplitude component shows a contrast similar to that of a conventional bright-field TEM image, which approximately represents the scattering contrast of the specimen. The amplitude component clearly shows the cubic shapes of the MgO particles, as some of the particles are schematically drawn in a wireframe representation on the right. There can be seen periodic contrast in the vacuum area. This contrast has the same interval as the pitch of the illumination beam shift and seems to be caused by the inaccurate recording of the hologram intensity. The inaccuracy of the recording could be caused by the nonlinear sensitivity of the recording camera, particularly in high-intensity regions of the holograms. Errors in the intensity of hologram could lead to errors in the reconstruction, particularly in vacuum regions where the reconstruction constraint based on the specimen interaction is small. Figure 7d shows the phase component of the reconstructed specimen transmission function, and Fig. 7e and f show the unwrapped phase profiles taken along lines (1) and (2) in Fig. 7d, respectively. The red dashed line in Fig. 7f shows a phase profile along line (2) after subtracting a background phase gradient obtained from the adjacent vacuum region. The linear variations of the phase along line (1) from 130 nm to 190 nm and from 200 nm to 310 nm correspond to the thickness change of the cubic particles with faces inclined about 45º toward the beam direction. The flat phase profile along line (2) from 70 nm to 120 nm and from 140 nm to 170 nm indicates a constant thickness region of the particles with faces oriented toward the beam direction. Mean inner potential of the MgO particle was determined to be 13.53±0.16 V from reconstructed phase change on the left particle along the line (2) in Fig. 7d. This value is close to the value reported in previous studies [22,23]. The reconstructed amplitude and phase of the specimen function are consistent with the results expected from the cubic shape of the MgO particles, thus indicating that the proposed method provides a reasonable reconstruction. Figure 7d also shows a phase gradient spreading around the MgO particles, as indicated by arrow (3) in the figure. This appears to be due to the electric field caused by the charging of the MgO particles since it exists mainly around the area where the MgO particles are aggregated. A steep phase shift is evident, as indicated by arrow (4) in Fig. 7d. This was caused by the internal potential of the supporting film.

## Concluding remarks

We propose a new configuration for electron near-field ptychography using full-field illumination and structured illumination using conductive film with openings. This method enables efficient and accurate phase measurement using a conventional TEM and camera with a small number of holograms, especially for large areas. The use of conductive film with openings enables the stable generation of a structured beam, which contributes to accurate reconstruction.

A simulation study showed a phase reconstruction with an error less than 1/3485 of the wavelength, which is comparable to that with other high precision phase measurement methods. An experimental study using MgO particles showed a phase reconstruction that was consistent with the cubic shapes of the particles. The reconstructed phase value for the MgO particle was equivalent to the mean inner potential of 13.53±0.16 V, which is close to the previously reported value, demonstrating reasonable reconstruction. In order to evaluate the reconstruction accuracy in detail, it is necessary to consider the influence of aberrations of the imaging optics, such as two-fold astigmatism. The aberrations alter the propagator in the reconstruction which is assumed to be free-space propagation in the current method. This could cause a discrepancy in the calculation of hologram formation and could increase reconstruction errors, especially for fine structures. These influences should be taken into account in observations at higher magnification. One potential solution for this issue is a numerical aberration correction in the reconstruction process. This should be investigated in future research.

The proposed method provides a means for quantitatively determining the electric field or inner potentials and the magnetic field or magnetic structure of materials. It is applicable to various materials such as semiconductors, batteries, and magnetic materials and is also applicable to soft-materials such as polymers and biological specimens, which have a meso-scale structure and is difficult to obtain sufficient image contrast with conventional imaging techniques. In addition, the proposed method is also beneficial for three-dimensional reconstruction since it enables efficient and robust observation of large areas and provides phase information that is proportional to the internal structure of the specimen. We are planning to apply the proposed method to depth-sectioning by independently reconstructing multiple planes and to super-resolution imaging by utilizing the structured illumination.

## References

[1] Gerchberg R W (1971) Phase determination from image and diffraction plane pictures in the electron microscope. *Optik* 34: 275–284.

[2] Miao J, Charalambous P, Kirz J, and Sayre D (1999) Extending the methodology of X-ray crystallography to allow imaging of micrometre-sized non-crystalline specimens. *Nature* 400: 342–344.

[3] Weierstall U, Chen Q, Spence J C H, Howells M R, Isaacson M, and Panepucci R R (2002) Image reconstruction from electron and X-ray diffraction patterns using iterative algorithms: experiment and simulation. *Ultramicroscopy* 90: 171–195. 10.1016/s0304-3991(01)00134-6 11942636

[4] Chapman H N, Barty A, Bogan M J, Boutet S, Frank M, Hau-Riege S P, Marchesini S, Woods B W, Bajt S, Benner W H, London R A, Plönjes E, Kuhlmann M, Treusch R, Düsterer S, Tschentscher T, Schneider J R, Spiller E, Möller T, Bostedt C, Hoener M, Shapiro D A, Hodgson K O, van der Spoel D, Burmeister F, Bergh M, Caleman C, Huldt G, Seibert M M, Maia F R N C, Lee R W, Szöke A, Timneanu N, and Hajdu J (2006) Femtosecond diffractive imaging with a soft-X-ray free-electron laser. *Nat. Phys*. 2: 839–843.

[5] Miao J, Ishikawa T, Robinson I K, and Murnane M M (2015) Beyond crystallography: diffractive imaging using coherent x-ray light sources. *Science* 348: 530–535. 25931551 10.1126/science.aaa1394

[6] Lo Y H, Zhao L, Gallagher-Jones M, Rana A, Lodico J J, Xiao W, Regan B C, and Miao J (2018) In situ coherent diffractive imaging. *Nat. Commun*. 9: 1826. 10.1038/s41467-018-04259-9 PMC5940918 29739941

[7] Huijts J, Fernandez S, Gauthier D, Kholodtsova M, Maghraoui A, Medjoubi K, Somogyi A, Boutu W, and Merdji H (2020) Broadband coherent diffractive imaging. *Nat. Photonics* 14: 618–622.

[8] Rodenburg J M and Faulkner H M L (2004) A phase retrieval algorithm for shifting illumination. *Appl. Phys. Lett*. 85: 4795–4797.

[9] Thibault P, Dierolf M, Menzel A, Bunk O, David C, and Pfeiffer F (2008) High-resolution scanning x-ray diffraction microscopy. *Science* 321: 379–382. 18635796 10.1126/science.1158573

[10] Rodenburg J M (2008) Ptychography and related diffractive imaging methods. *Adv. Imaging Electron Phys*. 150: 87–184.

[11] Faulkner H M L and Rodenburg J M (2005) Error tolerance of an iterative phase retrieval algorithm for moveable illumination microscopy. *Ultramicroscopy* 103: 153–164. 15774276 10.1016/j.ultramic.2004.11.006

[12] Sandberg R L, Paul A, Raymondson D A, Hädrich S, Gaudiosi D M, Holtsnider J, Tobey R I, Cohen O, Murnane M M, Kapteyn H C, Song C, Miao J, Liu Y, and Salmassi F (2007) Lensless diffractive imaging using tabletop coherent high-harmonic soft-x-ray beams. *Phys. Rev. Lett*. 99: 098103. 10.1103/PhysRevLett.99.098103 17931040

[13] Tate M W, Purohit P, Chamberlain D, Nguyen K X, Hovden R, Chang C S, Deb P, Turgut E, Heron J T, Schlom D G, Ralph D C, Fuchs G D, Shanks K S, Philipp H T, Muller D A, and Gruner S M (2016) High dynamic range pixel array detector for scanning transmission electron microscopy. *Microsc. Microanal*. 22: 237–249. 26750260 10.1017/S1431927615015664

[14] Stockmar M, Cloetens P, Zanette I, Enders B, Dierolf M, Pfeiffer F, and Thibault P (2013) Near-field ptychography: phase retrieval for inline holography using a structured illumination. *Sci. Rep*. 3: 1927. 10.1038/srep01927 PMC3668322 23722622

[15] Allars F, Lu P H, Kruth M, Dunin-Borkowski R E, Rodenburg J M, and Maiden A M (2021) Efficient large field of view electron phase imaging using near-field electron ptychography with a diffuser. *Ultramicroscopy* 231: 113257. 10.1016/j.ultramic.2021.113257 33773842

[16] Goodman J W (2005) *Introduction to Fourier Optics* (Roberts and Company Publishers, Greenwood Village, CO).

[17] Maiden A M and Rodenburg J M (2009) An improved ptychographical phase retrieval algorithm for diffractive imaging. *Ultramicroscopy* 109: 1256–1262. 19541420 10.1016/j.ultramic.2009.05.012

[18] Tonomura A (1999) *Electron Holography*. (Springer, Berlin, Heidelberg).

[19] Yamamoto K, Kawajiri I, Tanji T, Hibino M, and Hirayama T (2000) High precision phase-shifting electron holography. *J. Electron Microsc.* 49: 31–39. 10.1093/oxfordjournals.jmicro.a023789 10791418

[20] Clare R M, Stockmar M, Dierolf M, Zanette I, and Pfeiffer F (2015) Characterization of near-field ptychography. *Opt. Express* 23: 19728–19742. 26367630 10.1364/OE.23.019728

[21] Born M and Wolf E (2013) *Principles of Optics*. (Cambridge University Press, Cambridge).

[22] Yada K, Shibata K, and Hibi T (1973) A high resolution electron interference microscope and its application to the measurement of mean inner potential. *J. Electron Microsc*. 22: 223–230. 4776020

[23] Gajdardziska-Josifovska M, McCartney M R, De Ruijter W J, Smith D J, Weiss J K, and Zuo J M (1993) Accurate measurements of mean inner potential of crystal wedges using digital electron holograms. *Ultramicroscopy* 50: 285–299.

